# The feasibility of endoscopy-CT image registration in the head and neck without prospective endoscope tracking

**DOI:** 10.1371/journal.pone.0177886

**Published:** 2017-05-18

**Authors:** W. Scott Ingram, Jinzhong Yang, Beth M. Beadle, Richard Wendt, Arvind Rao, Xin A. Wang, Laurence E. Court

**Affiliations:** 1 Department of Radiation Physics, The University of Texas MD Anderson Cancer Center, Houston, Texas, United States of America; 2 The University of Texas Graduate School of Biomedical Sciences at Houston, Houston, Texas, United States of America; 3 Division of Radiation Oncology, The University of Texas MD Anderson Cancer Center, Houston, Texas, United States of America; 4 Department of Imaging Physics, The University of Texas MD Anderson Cancer Center, Houston, Texas, United States of America; 5 Department of Bioinformatics and Computational Biology, The University of Texas MD Anderson Cancer Center, Houston, Texas, United States of America; North Shore Long Island Jewish Health System, UNITED STATES

## Abstract

**Purpose:**

Endoscopic examinations are frequently-used procedures for patients with head and neck cancer undergoing radiotherapy, but radiation treatment plans are created on computed tomography (CT) scans. Image registration between endoscopic video and CT could be used to improve treatment planning and analysis of radiation-related normal tissue toxicity. The purpose of this study was to explore the feasibility of endoscopy-CT image registration without prospective physical tracking of the endoscope during the examination.

**Methods:**

A novel registration technique called Location Search was developed. This technique uses physical constraints on the endoscope’s view direction to search for the virtual endoscope coordinates that maximize the similarity between the endoscopic video frame and the virtual endoscopic image. Its performance was tested on phantom and patient images and compared to an established registration technique, Frame-To-Frame Tracking.

**Results:**

In phantoms, Location Search had average registration errors of 0.55 ± 0.60 cm for point measurements and 0.29 ± 0.15 cm for object surface measurements. Frame-To-Frame Tracking achieved similar results on some frames, but it failed on others due to the virtual endoscope becoming lost. This weakness was more pronounced in patients, where Frame-To-Frame tracking could not make it through the nasal cavity. On successful patient video frames, Location Search was able to find endoscope positions with an average distance of 0.98 ± 0.53 cm away from the ground truth positions. However, it failed on many frames due to false similarity matches caused by anatomical structural differences between the endoscopic video and the virtual endoscopic images.

**Conclusions:**

Endoscopy-CT image registration without prospective physical tracking of the endoscope is possible, but more development is required to achieve an accuracy suitable for clinical translation.

## 1. Introduction

Many head and neck cancers are visible by direct inspection, or more frequently, by the use of nasopharyngolaryngoscopy. The endoscopic examination is crucial for understanding the location and extent of disease prior to radiation treatment planning, since mucosal changes can indicate tumor involvement that may or may not correlate with the findings using cross-sectional imaging such as computed tomography (CT) or magnetic resonance imaging (MRI). Radiation oncologists who specialize in head and neck cancer frequently assess patients with endoscopy both at initial consultation and at follow-up for disease surveillance, and they also may utilize this technique during treatment to assess tumor response and radiation-related normal tissue toxicity, such as mucositis, edema, and ulceration.

Despite the frequent use of endoscopy, all spatial information depicting radiation dose is contained in the patient’s CT-based treatment plan. In order to use the endoscopic video quantitatively and objectively, the two modalities must be registered so that the information contained in the video can be mapped into CT space, i.e. the coordinate system of the CT scan. This additional information could be used to enhance tumor delineation during the treatment planning process and to provide new methods to analyze radiation-related normal tissue toxicity, which may not be appreciable using other imaging techniques.

Endoscopy-CT image registration is distinct from other forms of image registration used in radiation oncology because an endoscopic video frame is a 2D projection of 3D space, rather than a volumetric array. This disparity can be reduced by rendering virtual endoscopic images of the CT scan from the same coordinates, i.e. the same point of view, as the video frame. The virtual image itself thereby provides spatial correspondence between the pixels of the video frame and the voxels of the CT scan. In this scenario, the fundamental task of endoscopy-CT image registration is determining the CT-space coordinates at which to place the virtual endoscope.

One method of accomplishing this is to use electromagnetic sensors attached to the tip of the endoscope to physically track its coordinates. Electromagnetic tracking has been used in a variety of applications, including localization of the endoscope for image-guided endoscopic sinus surgery [1] and navigation for bronchoscopic biopsies [2]. Another option is to rely on image-based measurements to find the endoscope’s coordinates. With a rigid endoscope, such as those used for image-guided surgeries, this information can be inferred from the relative positions of infrared markers attached to the endoscope’s body [3]. However, routine clinical examinations of patients with head and neck cancer are performed with flexible endoscopes, and the position of the tip is not sufficiently constrained by the body to make use of external markers. In this case, the relative motion of the endoscope can be estimated frame-to-frame using point correspondences in adjacent frames of the video [4,5]. The image processing techniques used for these various approaches are an active area of research, including registration methods to estimate camera orientation [6,7] and image similarity metrics [8].

These prior initiatives had the goal of on-line guidance for endoscopic procedures, where real-time computing is critical. In these studies, determination of the endoscope’s CT-space coordinates was sufficient, and none evaluated the accuracy of using these coordinates to establish endoscopy-CT spatial correspondence. In contrast, registration and mapping can be done retrospectively for applications in radiotherapy, and establishing accurate spatial correspondence is the ultimate goal. Electromagnetic tracking has been used in this scenario to transfer contours of anatomical structures from video to CT [9] and to display CT-based radiation dose distributions on video frames [10]. However, the use of electromagnetic tracking for endoscopic examinations of patients with head and neck cancer requires a customized endoscope that is not available in most facilities. The patient must also lie down in exactly the same position used for the radiotherapy treatment planning CT, which is acquired with the patient’s head and shoulders secured in a rigid plastic mask. These requirements are not practical for routine outpatient endoscopic procedures, which are typically performed with the patient in a seated position. The purpose of this work is to explore the feasibility of an image-based approach to endoscopy-CT image registration that does not rely on prospective tracking during the endoscopic examination, and would ensure wider availability for treatment planning and toxicity analysis for patients with head and neck cancer.

## 2. Materials and methods

The process of endoscopy-CT image registration has three general steps:

1. Choose a video frame to be registered. In a clinical application, this would be a frame containing a structure of interest such as a tumor or an area of mucositis.
2. Find the endoscope’s CT-space coordinates in the selected frame. These coordinates consist of position and orientation.
3. Use the endoscope coordinates to establish spatial correspondence between the two modalities via virtual endoscopy.

The frame selected in step 1 will be referred to as the registration frame, and the coordinates found in step 2 will be referred to as the registered coordinates. In general, registered coordinates were found by maximizing the similarity between the registration frame and virtual endoscopic images, and video-frame pixel coordinates were mapped to CT-space using a calibrated pinhole model of the endoscope’s camera (sections 2.1 and 2.2).

The challenge is searching for the registered coordinates in an efficient and robust way. The prototypical method to do this is to track the endoscope across the recorded video by updating the coordinates of a virtual endoscope frame-to-frame, either by maximizing image similarity between the frame and the virtual image or by estimating motion based on point correspondences in the two images. However, this method requires registration of frames prior to the desired registration frame, and if the virtual endoscope becomes lost at any point in this process, the registration will fail without manual intervention. In this study, a novel technique to search for the registered endoscope coordinates that overcomes these limitations is presented (section 2.3.1), and its performance is evaluated and compared to frame-to-frame tracking on phantom and patient images (section 2.4).

### 2.1 Virtual endoscopy

Virtual endoscopic images were created by segmenting the luminal surface on CT, creating a triangular mesh from the resulting set of contours, and rendering images with a virtual camera placed inside the mesh. The luminal surface was segmented using a density threshold in the Pinnacle treatment planning software (Philips Healthcare). Virtual images were rendered with the Visualization Toolkit (VTK) [11] and the virtual camera was matched to the optical properties of the real endoscope described in section 2.4.1. These parameters include focal length, field of view, radial distortion, and principal point. They were obtained by selecting a set of frames from an endoscopic video viewing a checkerboard pattern and calibrating the camera with the calib3d module of OpenCV [12,13]. The scene lighting for the virtual endoscopic image is also an important consideration, and the simple lighting model available in VTK cannot match the properties of a real light source and human tissue. The endoscope’s image sensor has dynamic gain, which further complicates the scene lighting. However, it is possible to reproduce the large-scale variations in lighting with distance from the endoscope’s camera and across major edges in the scene. The lighting parameters used in this study were selected by visual inspection to match these characteristics, and they are summarized in Table 1. Descriptions of these parameters can be found at http://www.vtk.org/documentation/.

**Table 1:** Parameters of the VTK optical and lighting model.

Parameter	Value
RenderWindow size	720 x 486 pixels^2^
ViewAngle	50 degrees
LightType	Headlight
ConeAngle	50 degrees
AttenuationValues	1, 1.4, 2
Exponent	5
Intensity	20
AmbientColor	0, 0, 0
SpecularColor	0, 0, 0
DiffuseColor	1, 1, 1

Because the surface mesh is created from CT-space contours, the virtual image rendered at the registered coordinates allows video-frame pixels to be mapped to CT-space voxels. This is accomplished by projecting a ray through the pixel’s location in the virtual image and finding its intersection with the mesh. The focal length and field of view provided by the camera calibration allow this process to match the optics of the real camera.

### 2.2 Calculation of real-virtual image similarity

The similarity between the registration frame and the virtual endoscopic images was calculated using a combination of mutual information and intensity gradient alignment [14]. The endoscope has a position and an orientation in CT space, giving six degrees of freedom in its coordinates:

$$C=[\begin{array}{cccccc} x & y & z & \theta_{x} & \theta_{y} & \theta_{z} \end{array}]$$ (1)

Let *F* and *V*(*C*) denote an endoscopic video frame and the virtual image rendered at the coordinates *C*. The similarity measure is defined by

$$MI_{grad}(F,V(C))=MI(F,V(C))∙\sum_{\left(f,v)\in (F,V(C)\right)}\frac{\cos(\phi_{f,v})+1}{2}∙\min\left(\left|\nabla F(f)|,|\nabla V(v)\right|\right)$$ (2)

In this equation *MI(F*, *V(C))* is the mutual information of the frame and the virtual image. It is weighted by a sum over all pairs of corresponding pixels *(f*, *v)* in the two images. In this sum, *ϕ*_*f*,*v*_ is the angle between the intensity gradients of the two images, and |∇*F(f)*| and |∇*V(v)*| are the magnitudes of the intensity gradients of the two images. This weighting factor favors real-virtual image pairs that have edges in the same location and direction. Using Eqs 1 and 2, the registered coordinates for a registration frame *F* are defined by

$$C_{reg}\equiv {\mathrm{argmax}}_{C\in R^{6}}\left(MI_{grad}(F,V(C))\right)$$ (3)

### 2.3 Techniques to search for registered endoscope coordinates

Once a registration frame has been selected, the next step is to find the endoscope’s registered coordinates *C*_*reg*_. A brute-force search of the entire 6D coordinate space would require a prohibitive amount of computation time. However, in practice the endoscope’s motion is constrained by its coordinates in previous frames and by physical considerations such as the insertion direction and the operator’s hand, which determines the roll angle. Two techniques were tested in this study that take advantage of these constraints to reduce the size of the search space in Eq 3 and to search for *C*_*reg*_ efficiently. The first is the previously-established frame-to-frame tracking method. The second is a novel technique developed to overcome the limitations of frame-to-frame tracking. These techniques are described in general detail in sections 2.3.1 and 2.3.2. Application-specific details for phantom and patient images are described in sections 2.4.2 and 2.4.3.

#### 2.3.1 Frame-To-Frame Tracking

In this technique, the virtual endoscope is repeatedly moved such that the virtual image matches the next frame in the video. It consists of the following steps:

1. Select a registration frame *F*_*reg*_.
2. Select a starting frame *F*_*0*_ and place the virtual endoscope at the corresponding coordinates *C*_*0*_ by visually aligning anatomical structures or by mathematically aligning point correspondences.
3. Get the next frame *F*_*1*_ and use *C*_*0*_ as the initial guess to search for the coordinates *C*_*1*_ that maximize the similarity between *F*_*1*_ and the virtual endoscopic image *V(C)*. In this study the Nelder-Mead simplex method was used to search the coordinate space [15].
4. Repeat step 3 for frame *F*_*2*_ using *C*_*1*_ as the initial guess, and then for frame *F*_*3*_ using the resulting *C*_*2*_ as the initial guess, and so on until *F*_*reg*_ is reached.

Frame-To-Frame Tracking is straightforward, but the results at each frame depend on the results of the previous frame. The Nelder-Mead simplex method can find only the nearest local minimum, so if the virtual endoscope gets off track at some point and becomes lost, the process fails.

#### 2.3.2 Location Search

In any endoscopic video there are likely to be many scenarios that may cause Frame-To-Frame Tracking to fail, including lighting changes from the endoscope’s dynamic gain, transient muscle motion causing large structural changes, erratic camera motion, and blurry frames. But Frame-To-Frame Tracking requires determination of the coordinates of the endoscope for all frames from *F*_*0*_ up to *F*_*reg*_, even though only the coordinates *C*_*reg*_ are of interest, and the longer this sequence of frames, the greater the chance of encountering an impasse. A more robust method would be to search directly for the desired frame’s coordinates without considering any frames before it, and that is the goal of the Location Search technique developed for this study.

**Fig 1:**
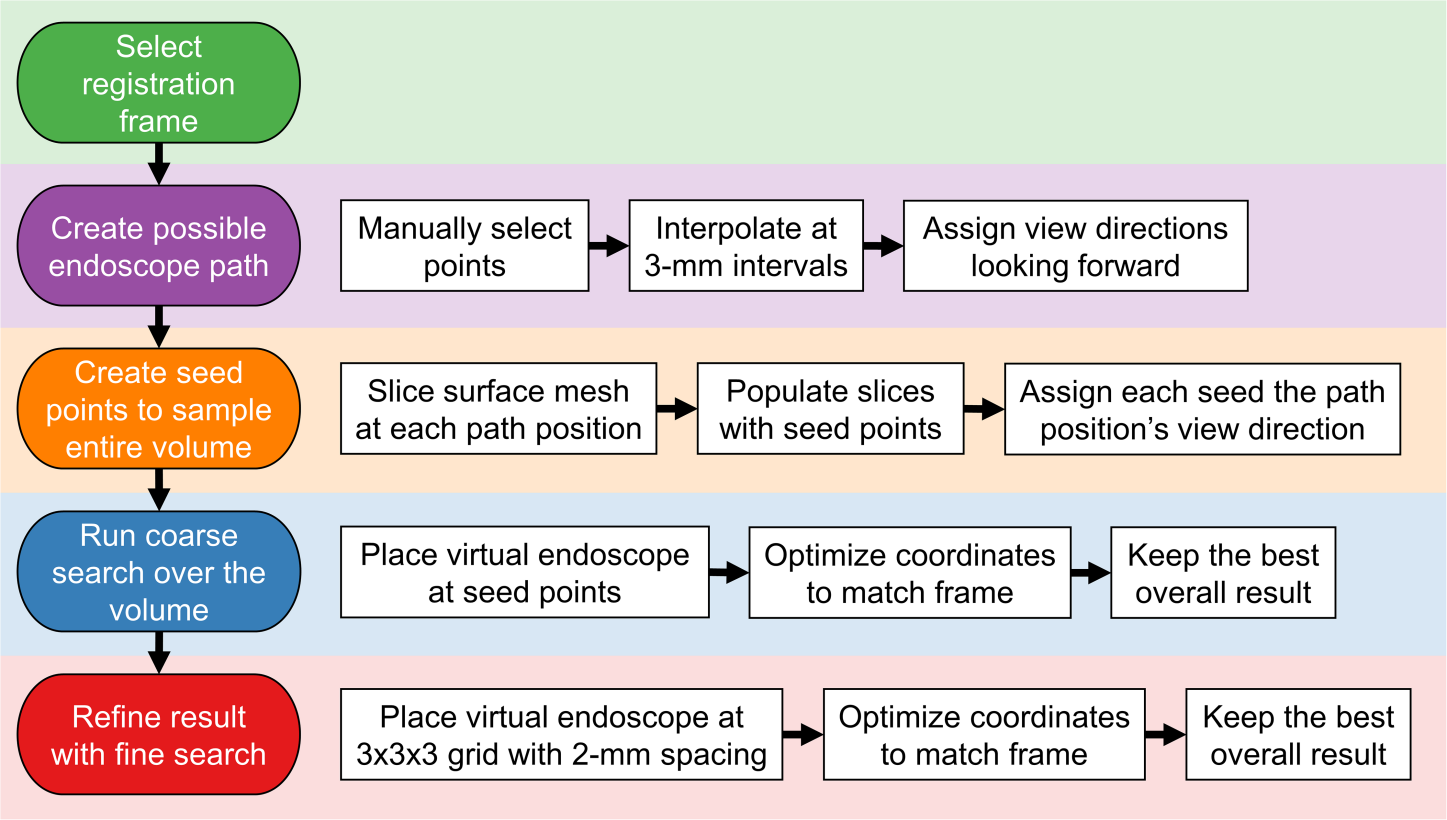
The Location Search technique. A flowchart showing the steps of the Location Search registration technique.

This technique takes advantage of the physical constraints on the endoscope’s view direction to reduce the search space and perform a coarse search of the entire volume that places the virtual endoscope near the desired location. This result is refined with a local search to obtain the final coordinates *C*_*reg*_. It consists of the following steps, which are also illustrated as a flowchart in Fig 1:

1. Select a registration frame *F*_*reg*_.
2. Create a possible path through the volume for the virtual endoscope.
  - Manually select a small set of points covering the length that the endoscope can travel.
  - Interpolate between these points at evenly-spaced intervals. In this study, an interval of 3 mm was used.
  - Assign view directions to each point such that the virtual endoscope looks at the next point.
3. At each path point, create a set of seed points that samples the cross-sectional area of the surface mesh.
  - Slice the surface mesh in the plane perpendicular to the virtual endoscope’s view direction.
  - Calculate the desired number of seed points *S* by dividing the slice’s cross-sectional area by the real endoscope’s cross-sectional area. This creates seed points that are 0.3–0.4 cm apart.
  - Use k-means clustering with *S* clusters to generate the seed points within the slice’s cross-sectional area.
  - Assign to each seed point the same view direction as that of the path point from which the slice was created.
4. Perform the coarse search by starting from each seed point in each slice and searching for the virtual endoscope coordinates that maximize the similarity between *F*_*reg*_ and the virtual image *V(C)*. In this step the virtual endoscope’s position [*x y z*] is fixed and the Nelder-Mead simplex is used to search for the view direction [*θ*_*x*_ *θ*_*y*_ *θ*_*z*_].
5. Create a 3 x 3 x 3 grid of points with 0.2-cm spacing centered on the best overall result from step 4. Assign each grid point the same view direction as this result.
6. Perform the fine search by starting from each grid point and searching for the virtual endoscope coordinates that maximize the similarity between *F*_*reg*_ and the virtual image *V(C)*. In this step all six coordinates [*x y z θ*_*x*_ *θ*_*y*_ *θ*_*z*_] are optimized using the Nelder-Mead simplex.

Location Search is more complex than Frame-To-Frame Tracking, but the results for a given frame do not depend on any frames before or after, and manual input is required only for the selection of the initial path points in step 2. This selection is a simple process because it does not matter if the selected path is the same as the actual path the endoscope takes in the recorded video. The path is only used to initialize the seed point view directions to be reasonably close to what can be expected at any given location in the anatomy. Step 2a is illustrated in more detail in sections 2.4.2 and 2.4.3.

**Fig 2:**
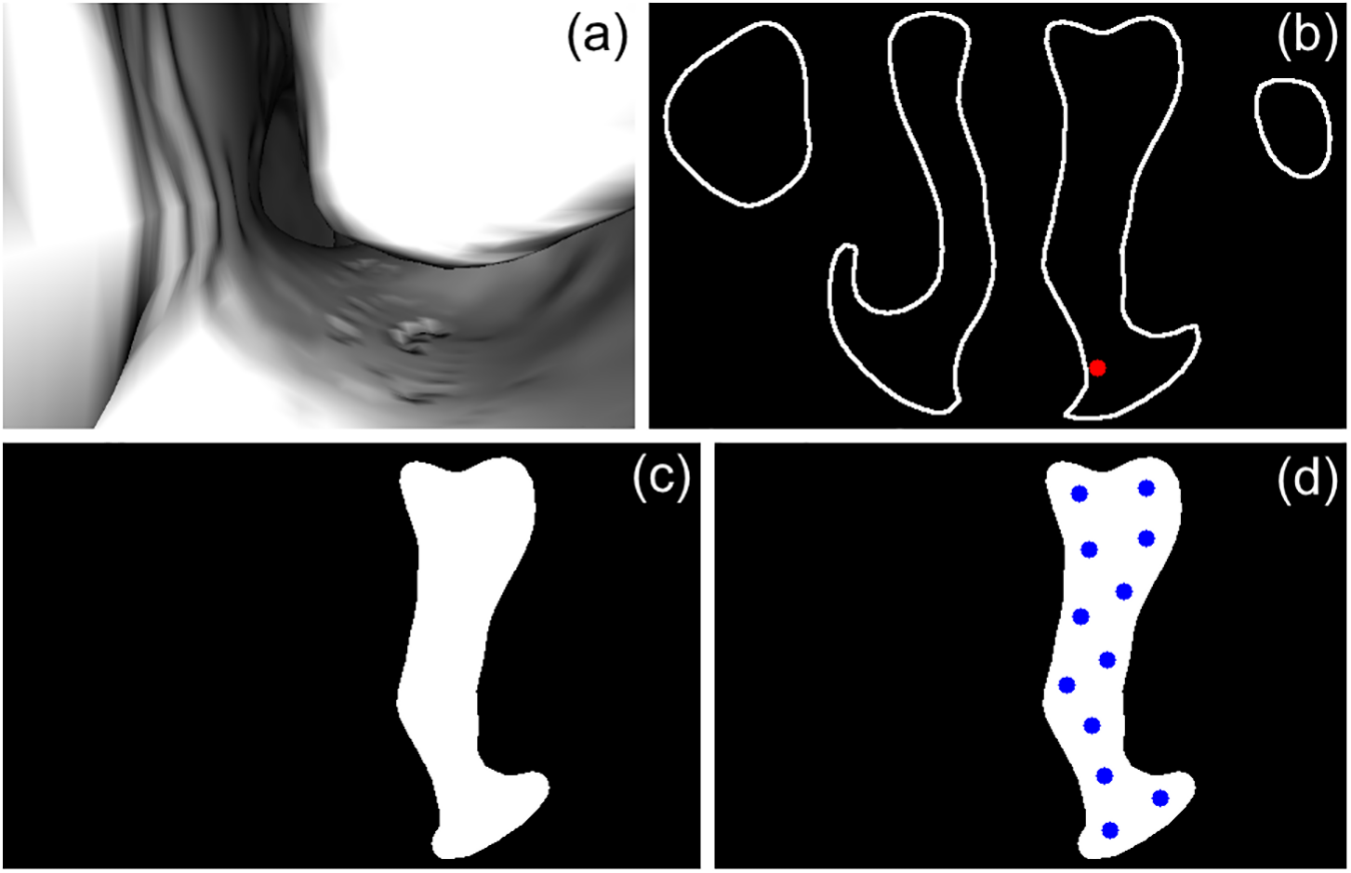
Slicing the surface mesh in step 3 of Location Search. (a) The virtual endoscopic image at a path position in the left side of the nasal cavity looking towards the pharynx. On the left is the medial wall of the nasal cavity, and on the right is the inferior concha. (b) The rendered image of the slice outline. Both sides of the nasal cavity can be seen, as well as the maxillary sinuses. The virtual endoscope’s position is shown as a red dot. (c) The outline has been flood-filled from the virtual endoscope’s position and unconnected regions have been discarded. (d) The centroids of the k-means clusters are shown as blue dots. This image was cropped slightly to fit the template.

Step 3 is illustrated in Fig 2, and it requires more explanation of the computational details. The slice is generated by using VTK methods to define a plane perpendicular to the virtual endoscope’s view direction and render an image of the intersection of this plane with the surface mesh. The extent of the points in this intersection is used to calculate the distance behind the virtual endoscope from which the image must be rendered in order to contain the entire slice. This creates an image of the outline of the slice, which is flood-filled from the virtual endoscope position. Any non-filled regions are discarded to eliminate unnecessary seed points in unconnected regions, such as the contralateral nasal cavity. Each pixel [*u*, *v*] in the filled slice is treated as an individual observation to partition with k-means clustering, and the centroid of each cluster is converted to a 3D point in CT-space, giving the desired seed points for the slice. This 2D-to-3D conversion is possible because the rendering camera’s position and orientation are known, as well as the distance from the camera to the pixels in the slice.

In order to prevent under-sampling of large slices and over-sampling of small ones, the number of seed points must vary between slices. This number is calculated in Step 3b by dividing the area of the slice by the cross-sectional area of the endoscope, resulting in seed points spaced 0.3–0.4 cm apart. There is no exact requirement for the spacing or number of seed points, only that the volume be sampled densely enough that the virtual endoscope gets a successful match to the registration frame. If there are too few seed points, the sparse sampling may prevent this from happening. The outcome of steps 3 and 4 is refined with a dense local search in steps 5 and 6, so any increase in the number of seed points beyond the sufficient minimum will increase computation time with no substantial improvement in the results. However, there is no way to calculate what the sufficient minimum is, so division by the endoscope’s cross-sectional area was chosen on physical grounds as a reasonable starting point at the outset of this study.

### 2.4 Testing the performance of Frame-To-Frame Tracking and Location Search

#### 2.4.1 Endoscopic videos and image preprocessing

Endoscopic videos were recorded using an Olympus ENF-VQ rhinolaryngoscope (Olympus America, Center Valley, PA), which is a flexible video endoscope with a working length of 30 cm and an outer diameter of 0.4 cm. The endoscope’s output was recorded as 720 x 486 pixels^2^ MPEG-2 at 30 frames per second using the nStream G3 video recorder (Image Stream Medical, Littleton, MA). All video frames were converted to grayscale and radial distortion was removed using the calibration parameters described in section 2.1.

For calculations of image similarity, the video frames and virtual endoscopic images were smoothed and downsampled to improve performance and speed. Video frames were smoothed with a 3 x 3 Gaussian of σ = 1, and virtual images were smoothed with a 9 x 9 Gaussian of σ = 3. Video frames were smoothed less because they are inherently more blurry than the virtual images, so the smallest degree of smoothing that was sufficient to remove the MPEG compression artifacts was chosen. All images were downsampled by a factor of 4 to 180 x 121 pixels^2^.

#### 2.4.2 Phantoms

**Fig 3:**
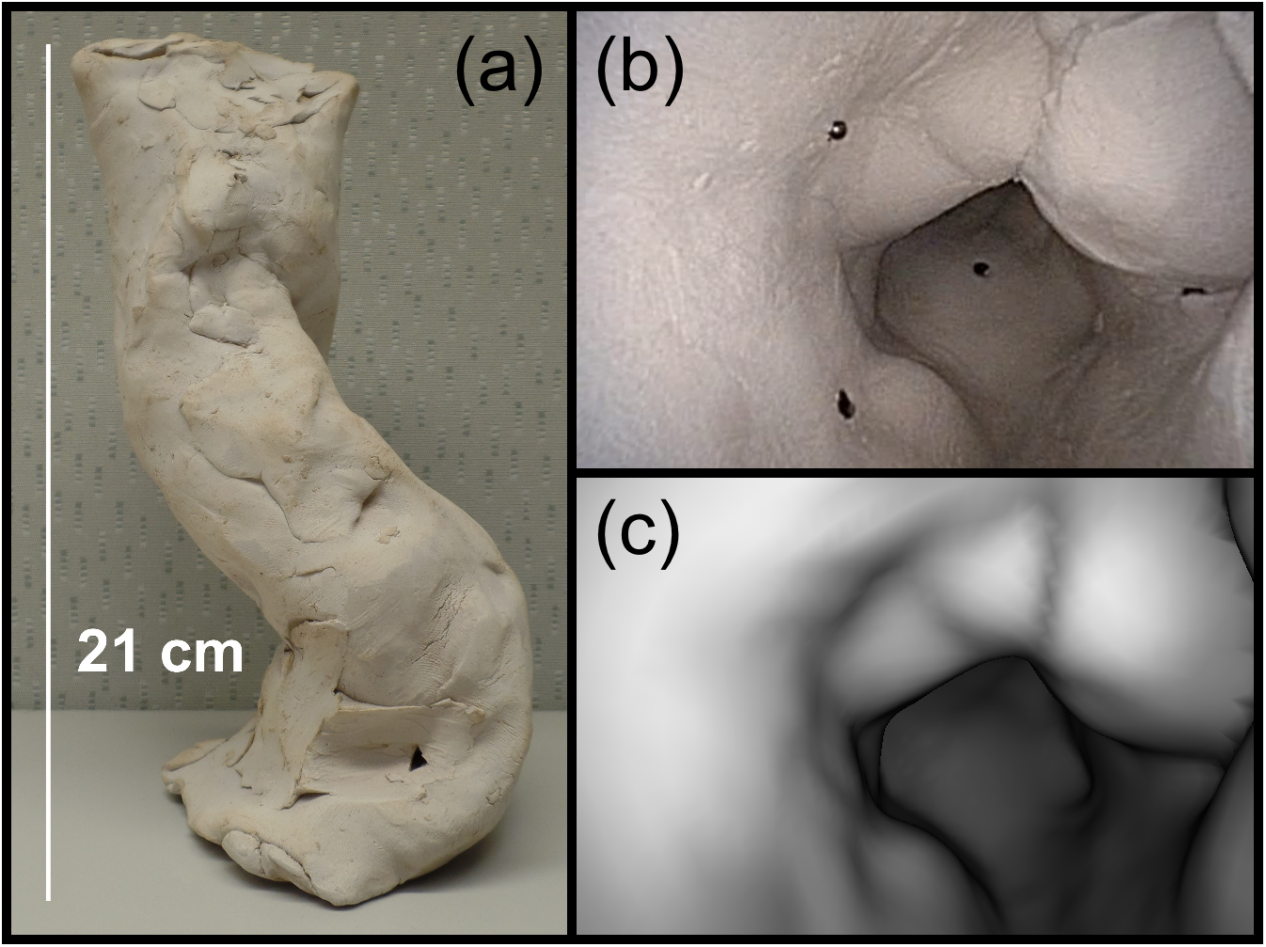
The clay phantom with radiopaque markers. (a) A picture of the marker phantom. The inner diameter is about 5 cm. (b) An endoscopic video frame from inside the phantom with four of the markers visible. (c) The corresponding virtual endoscopic image.

**Fig 4:**
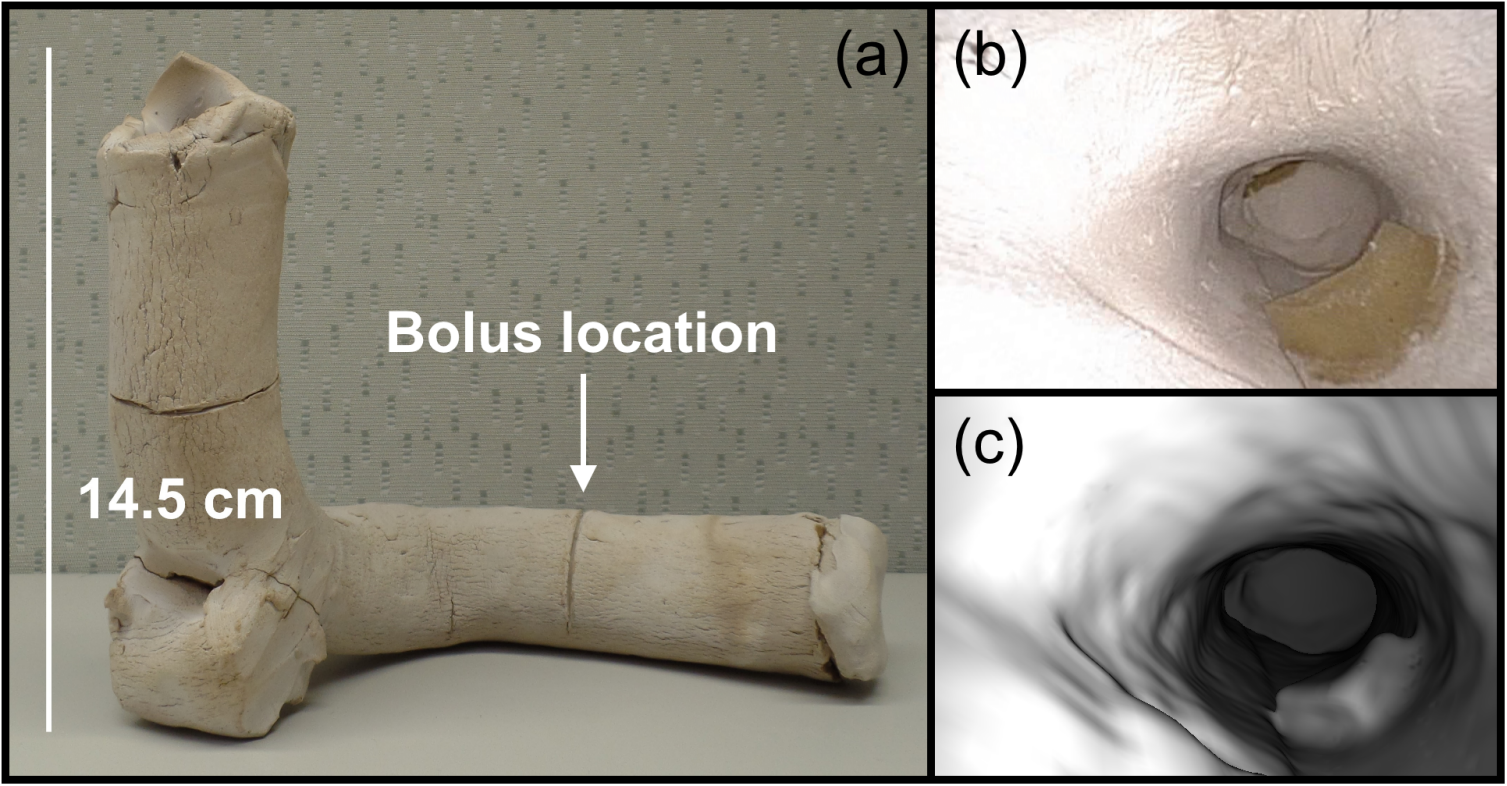
The clay phantom with bolus material. (a) A picture of the bolus phantom. This phantom is smaller than the marker phantom, with an inner diameter of about 2 cm. (b) An endoscopic video frame from inside the phantom with the bolus visible on the lower right. (c) The corresponding virtual endoscopic image.

Two clay phantoms were created to assess the performance of the registration techniques. Clay was used so that the phantoms would have irregular shapes and low-contrast surface textures. The first phantom contains twelve 0.2-cm-diameter radiopaque markers embedded in the luminal surface (Fig 3) and the second phantom contains a 1 x 1 x 0.5 cm^3^ piece of Superflab (Mick Radio-Nuclear Instruments, Mount Vernon, NY), which is a bolus material that is used to match the radiation attenuation properties of human tissue (Fig 4). CT scans of the phantoms were acquired using a Lightspeed RT (GE Healthcare) with a 30-cm field of view and 1-mm slices. The luminal surface of each phantom was segmented on CT using a density threshold of 0.9 g/cm^3^ to create the surface meshes for virtual endoscopy.

In each phantom, two video sequences were recorded with the endoscope moving through the length of the phantom. The lengths of the video sequences were 27 and 17 seconds in the marker phantom and 53 and 75 seconds in the bolus phantom, with the bolus visible for the last 42 and 64 seconds, respectively. The bolus phantom videos were longer due to the increased difficulty of navigating the endoscope through the smaller space. A set of registration frames was selected for each phantom by sampling the videos at regular intervals (1 and 2 seconds for the marker and bolus phantoms, respectively) and identifying the least blurry frame out of the sample and the five previous and subsequent frames. The least blurry frame was identified as the one with the largest variance after filtering with a 3 x 3 Laplacian. The endoscopic videos contained many frames that were unsuitable for registration, either due to the markers or bolus not being visible or due to under- or overexposure as the image sensor adjusted its gain. To avoid these scenarios, each set of frames was reviewed and unsuitable frames were rejected. This resulted in a total of 36 and 37 registration frames for the marker and bolus phantoms, respectively.

Both registration techniques were tested on these frame sets. Frame-To-Frame Tracking was run as described in section 2.3.1. The initial virtual endoscope coordinates *C*_0_ were established by mathematically aligning the pixel locations of manually-selected point correspondences in the starting frame *F*_0_ and a nearby virtual endoscopic image; this process is known as resectioning [12]. A total of 775 and 457 frames were traversed for the marker phantom video sequences, and 1535 and 2186 frames were traversed for the bolus phantom video sequences.

**Fig 5:**
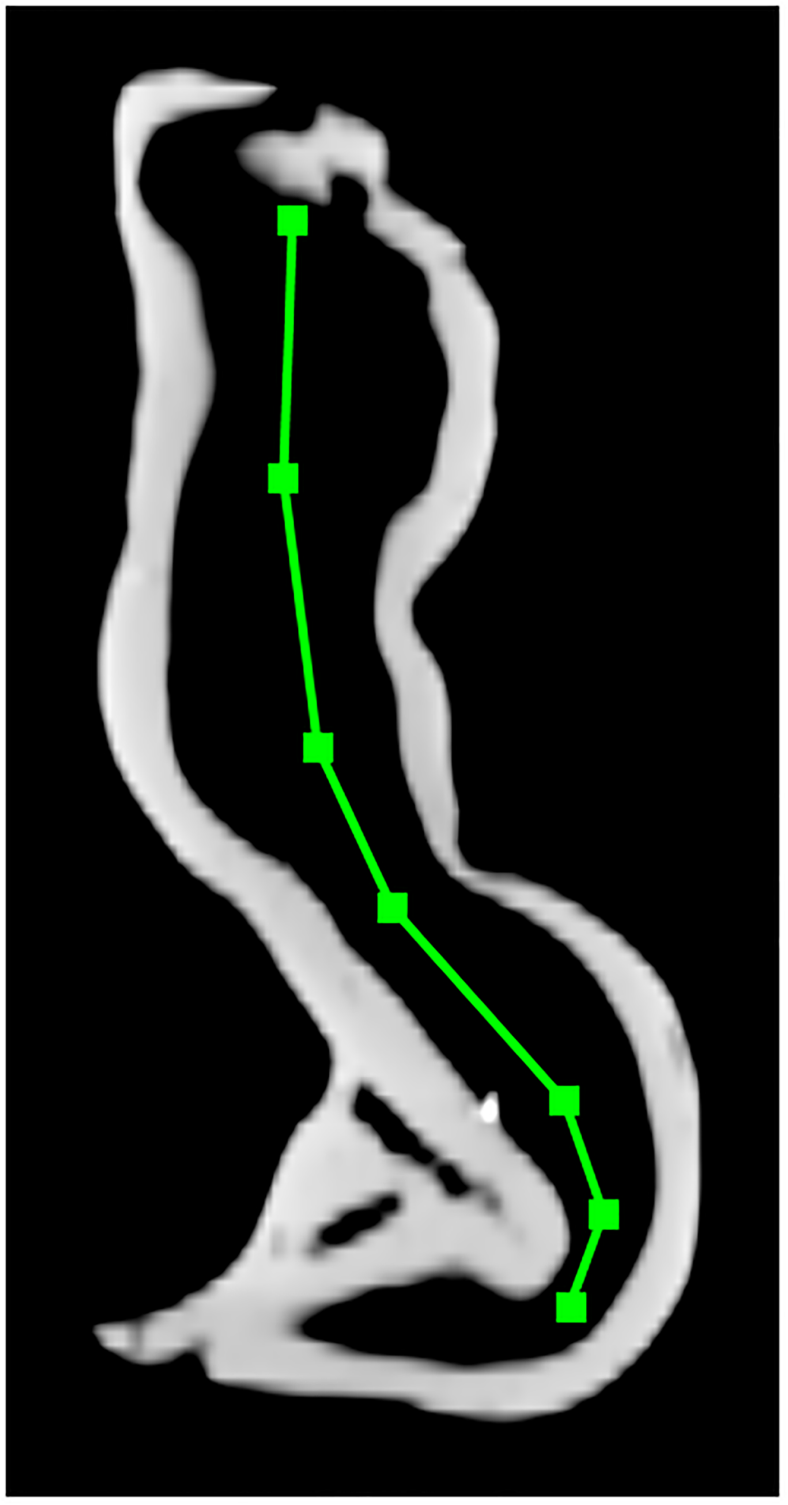
Virtual endoscope path creation in the marker phantom. A coronal CT slice shows the manually-selected virtual endoscope path through the marker phantom used for the Location Search registration technique.

For Location Search, the endoscope path in step 2 (section 2.3.2) was created differently for the two phantoms. The marker phantom path was created by manually selecting seven points on a coronal slice of the CT (Fig 5). The bolus phantom does not have bilateral symmetry, so instead of manual selection, the centroid of the segmented surface was calculated for every fifth CT slice. Location Search was run as described in section 2.3.2, with the exception of calculating the number of seed points in each slice for the marker phantom (step 3b). The marker phantom has larger dimensions, and hence larger distances between the endoscope and the surface, so a given change in the virtual endoscope’s position produces a smaller change in the virtual image. For this reason, the number of seed points per unit slice area was reduced to avoid excessive computation times. This resulted in seed points spaced 0.6–0.8 cm apart rather than 0.3–0.4 cm.

The outputs of Frame-To-Frame Tracking and Location Search are sets of registered coordinates *C*_*reg*_ for each registration frame *F*_*reg*_ in the test sets for both phantoms. These coordinates were used to test registration accuracy by projecting pixel locations in *F*_*reg*_ into the surface mesh using the virtual image rendered at *C*_*reg*_.

**Fig 6:**
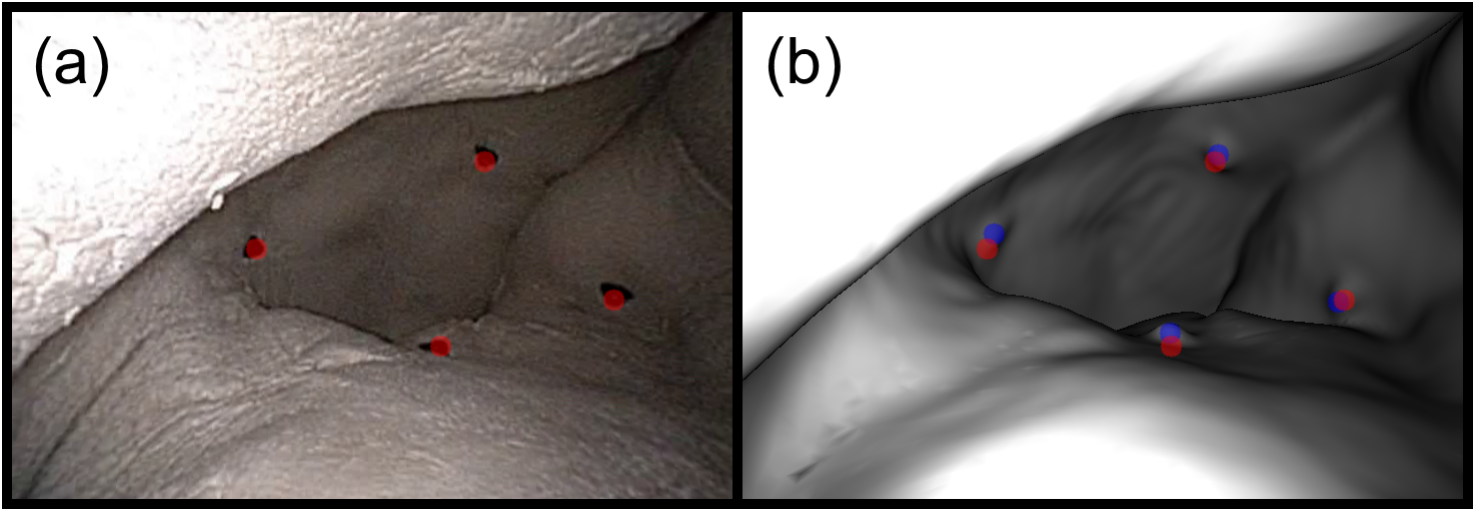
The measurement of CT-space marker positions. (a) A video frame with four markers visible. Their manually-selected pixel locations are overlaid in red. (b) The corresponding virtual endoscopic image rendered at the registered coordinates. The manually-selected pixel locations are overlaid in red and the image-plane projections of their true 3D locations are overlaid in blue. The average 3D error for these four measurements was 0.29 cm, and the average 2D error was 11 pixels.

The marker phantom was used to evaluate the performance of the two techniques for point measurements (see Fig 6). First, the pixel locations of any markers visible in each registration frame were selected manually. Next, these pixel locations were projected into the surface mesh to take 3D measurements using the virtual image rendered at the registered coordinates. Finally, the true 3D locations of the markers were selected manually on CT and the 3D distances between the true and measured locations were calculated. One to six markers were visible in each frame and a total of 126 measurements were taken.

**Fig 7:**
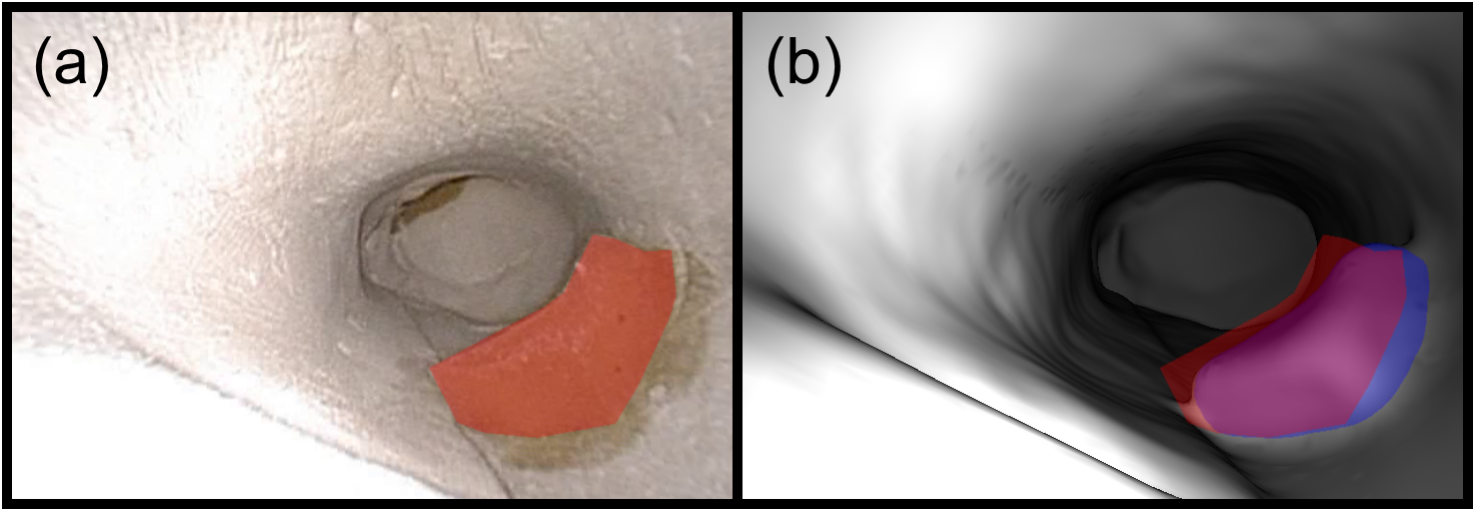
The measurement of the CT-space bolus surface. (a) A video frame showing the bolus material with the manually-drawn contour overlaid in red. The contour contains over 28,000 pixels, but downsampling reduced this to 613 pixels spaced evenly within the area. (b) The corresponding virtual endoscopic image rendered at the registered coordinates. The manually-drawn contour is overlaid in red and the image-plane projection of the bolus volume is overlaid in blue. The MAD for this frame was 0.23 cm and the Dice coefficient between the 2D contours was 0.82.

The bolus phantom was used to evaluate the performance of the two techniques for object measurements (see Fig 7). First, the CT voxels representing the surface of the bolus were identified (n = 612). Next, the bolus was manually contoured in each registration frame by selecting a set of points around its perimeter, and a binary mask of this contour was created. This mask was downsampled such that the number of pixels inside the contour was approximately equal to the number of bolus surface voxels. Then, each pixel in the mask was projected into the surface mesh to take 3D measurements using the virtual image rendered at the registered coordinates (604 ≤ n ≤ 622). Finally, the mean absolute distance (MAD) was calculated between the bolus surface voxels and the measured locations according to the following equation:

$$Mean\;absolute\;distance=\frac{1}{\left(n_{M}+n_{CT}\right)}\left(\sum_{i=1}^{n_{M}}d_{i}^{M\to CT}+\sum_{j=1}^{n_{CT}}d_{j}^{CT\to M}\right)$$ (4)

In this equation, *n*_*M*_ is the number of pixels in the bolus contour mask and *n*_*CT*_ is the number of bolus surface voxels. The term $d_{i}^{M\to CT}$ is the minimum distance from the i^th^ projected mask pixel to any surface voxel, and $d_{j}^{CT\to M}$ is the minimum distance from the j^th^ surface voxel to any projected mask pixel.

#### 2.4.3 Patients

After approval from the Institutional Review Board, three radiotherapy patients with head and neck cancer were enrolled on a protocol allowing their routinely-obtained endoscopic examinations to be archived for this study. Informed consent was obtained in writing prior to enrolling each patient. For patient 1, two examinations were recorded during and at the end of the course of radiotherapy, 28 and 49 days after the planning CT was acquired. For patients 2 and 3, a single examination was recorded one day after the planning CT was acquired. The planning CTs were all acquired using a Brilliance 64 (Philips Healthcare) with a 50-cm field of view and 0.1-cm slices thickness. The planning CTs were interpolated to a 30-cm field of view before segmenting with a density threshold of 0.6 g/cm^3^ to create the surface meshes for virtual endoscopy. This interpolation was done to increase the number of triangles in the meshes, which improves structural resolution.

At this point in the study, patient 2 was excluded from further analysis. This was done because the patient had a large base-of-tongue tumor that obstructed much of the airway in the oro- and hypopharynx. This obstruction caused the surface mesh to lose most of the characteristic anatomical structure that allows the similarity measure to match virtual endoscopic images to video frames. The large size of the tumor also caused most of the video to be close-up views of the surface, for which virtual endoscopy can reproduce neither the texture nor the specular reflections from fine structural details that are not present in the surface mesh.

The video sequences were 66 and 48 seconds long for patient 1 and 64 seconds for patient 3. A set of registration frames was selected for each patient using the method described in section 2.4.2 with two-second sampling intervals. Frames that were unsuitable due to poor lighting, close-up views, or transient muscle motion were manually identified and rejected, leaving a total of 38 and 23 registration frames for patient 1 and patient 3, respectively.

**Fig 8:**
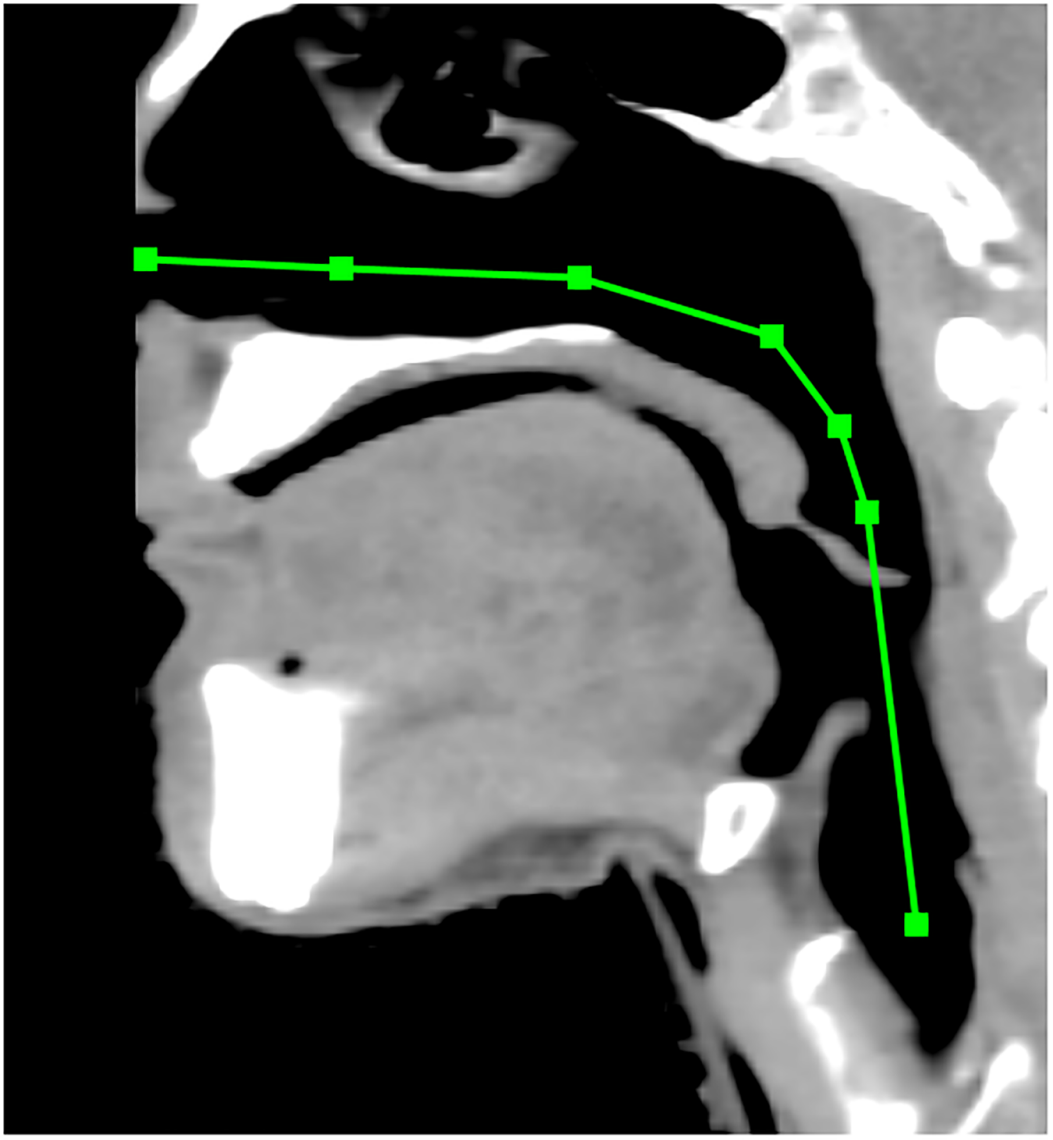
Virtual endoscope path creation in a patient. A sagittal CT slice shows the manually-selected virtual endoscope path through a patient used for the Location Search registration technique. The nose is cropped out due to the field-of-view reduction described in section 2.4.3.

Both registration techniques were tested on these frame sets. Frame-To-Frame Tracking was run as described in section 2.3.1. The initial virtual endoscope coordinates *C*_0_ were established in the nasal cavity by manually placing the virtual endoscope to visually match the initial frame. A total of 1975 and 1202 frames were traversed for patient 1’s video sequences, and 1679 frames were traversed for patient 3’s video sequence. Location Search was run as described in in section 2.3.2. The endoscope path in step 2 was created by manually selecting a set of points on a sagittal slice of the planning CT (see Fig 8).

**Fig 9:**
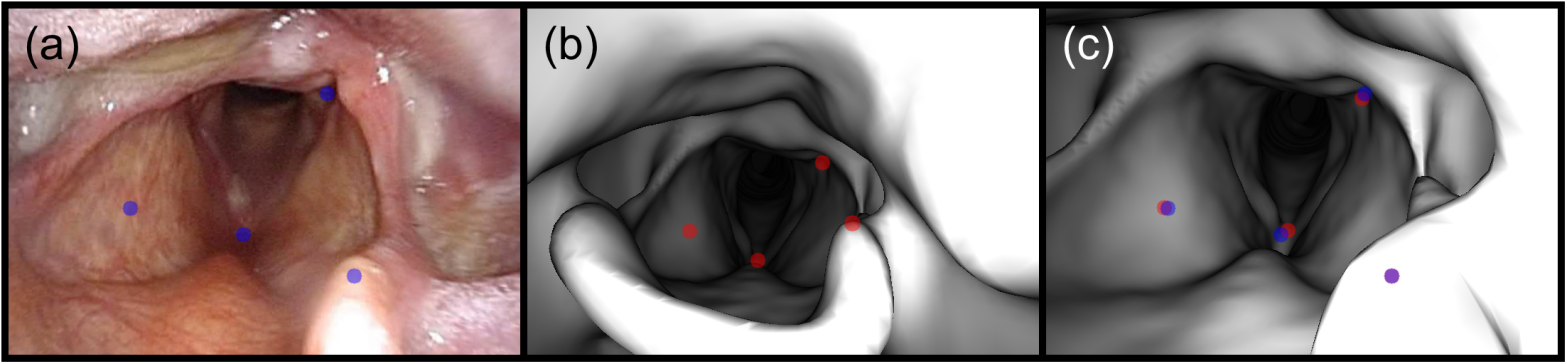
Obtaining ground-truth endoscope coordinates via camera resectioning. (a) A video frame showing the epiglottis and glottis with manually-selected points overlaid in blue. (b) A virtual endoscopic image rendered at the initial guess for the endoscope coordinates with the manually-selected corresponding points overlaid in red. These points are projected into the surface mesh to get their 3D positions. (c) The virtual endoscopic image rendered at the ground-truth endoscope coordinates. These coordinates are obtained by moving the virtual endoscope to minimize the pixel distances between the video frame points (overlaid in blue) and the image-plane projections of their 3D coordinates (overlaid in red).

As with the phantoms, the outputs of Frame-To-Frame Tracking and Location Search are sets of registered coordinates *C*_*reg*_ for each registration frame *F*_*reg*_ in the test sets for both patients. Unlike the phantoms, the patient images do not have fiducial objects, so the registration accuracy was tested by comparing the registered coordinates to ground-truth coordinates obtained by camera resectioning. This was accomplished by placing the virtual endoscope such that manually-selected point correspondences could be established between the virtual image and the registration frame. 3D coordinates were obtained by projecting the virtual image points into the surface mesh, and least-squares minimization was used to move the virtual camera such that these 3D points were projected onto the image plane as close as possible to their expected locations in the registration frame. This process is illustrated in Fig 9.

## 3. Results

### 3.1 Marker phantom

**Fig 10:**
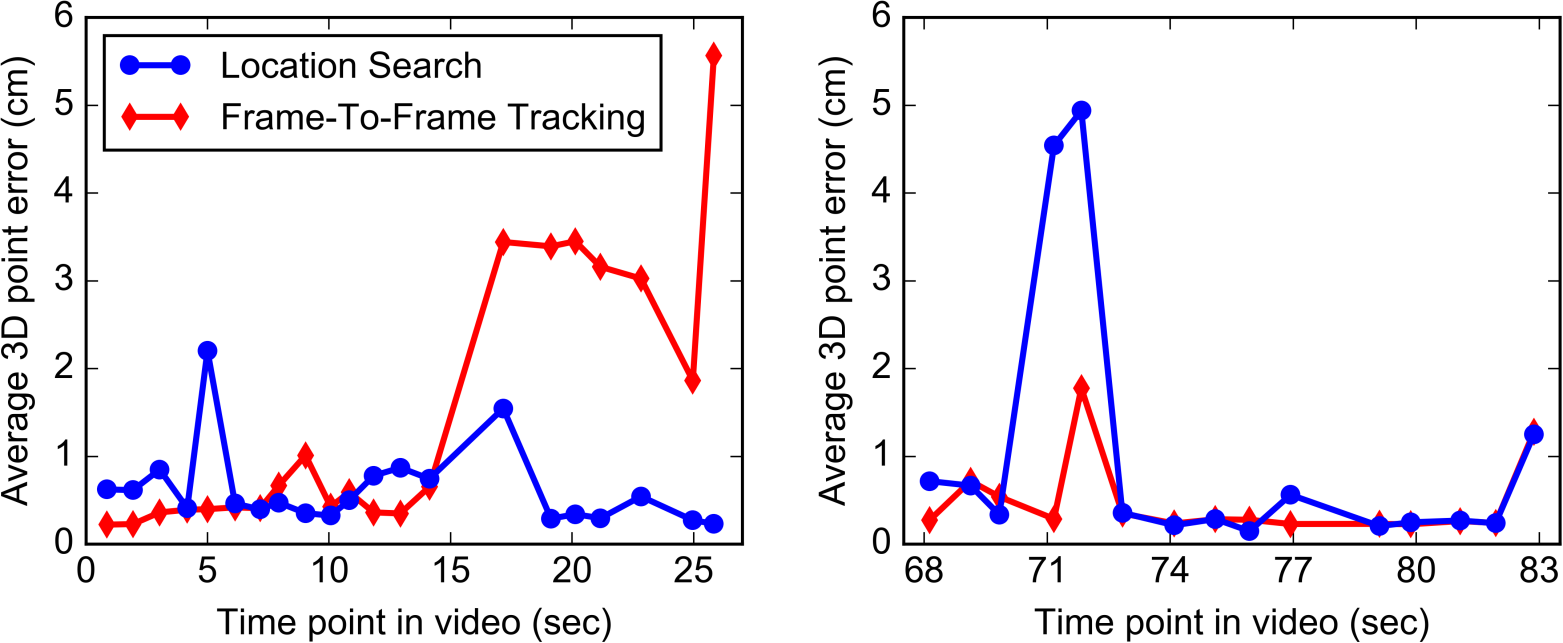
Results of the two registration techniques in the marker phantom. The average 3D point errors for each registration frame in video sequence 1 (left) and video sequence 2 (right) are shown for both registration techniques vs. the frames’ time points in the video. In sequence 1, the virtual endoscope became lost during Frame-To-Frame Tracking, resulting in very large errors for the last seven frames. In sequence 2, Location Search failed on two frames, resulting in very large errors.

A comparison of the registration accuracy of Frame-To-Frame Tracking and Location Search in the marker phantom is shown in Fig 10.

#### 3.1.1 Frame-To-Frame Tracking

The average 3D point error over all registration frames was 1.06 ± 1.48 cm, and the median error was 0.35 cm. The large average and standard deviation were due in part to a difference in performance between the two video sequences, with average errors of 1.36 ± 1.69 cm for sequence 1 and 0.48 ± 0.59 cm for sequence 2. The individual median errors were 0.40 and 0.28 cm, respectively. The difference is due to the fact that the virtual endoscope became lost about two thirds of the way through video sequence 1, resulting in very large errors for the last seven registration frames (see Fig 10). Omitting these frames reduces the average error for video sequence 1 to 0.46 ± 0.45 cm and the median error to 0.30 cm, and the overall average and median errors to 0.47 ± 0.51 cm and 0.29 cm, respectively.

#### 3.1.2 Location Search

The average 3D point error over all registration frames was 0.68 ± 0.95 cm, and the median error was 0.36 cm. The performance was similar for both video sequences, although the technique failed on two registration frames in sequence 2, resulting in very large errors for four of the 126 point measurements (see Fig 10). Omitting these two frames reduces the overall average error to 0.55 ± 0.60 cm and the median error to 0.34 cm, both of which are somewhat larger than the reduced errors obtained using Frame-To-Frame Tracking.

### 3.2 Bolus phantom

**Fig 11:**
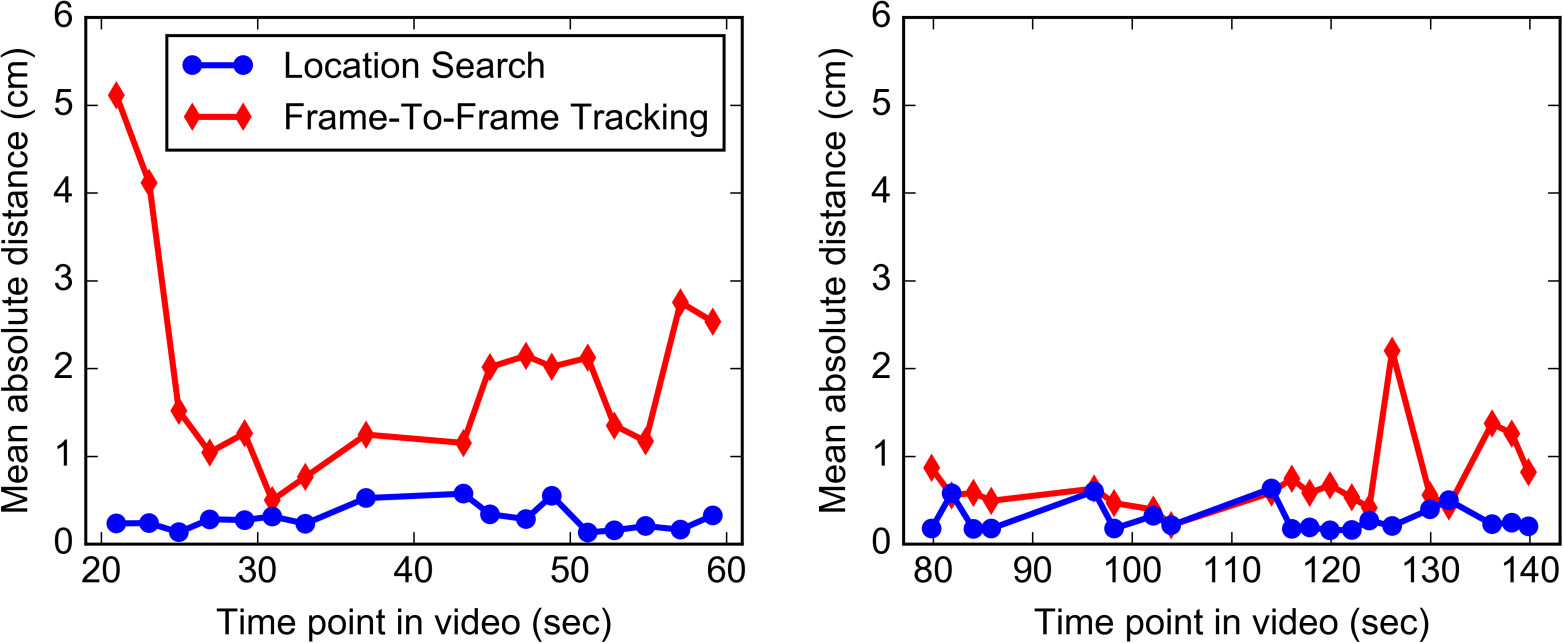
Results of the two registration techniques in the bolus phantom. The mean absolute distances (MADs) between the measured and ground truth bolus contours for each registration frame in video sequence 1 (left) and video sequence 2 (right) are shown for both registration techniques vs. the frames’ time points in the video. Location Search performed better than Frame-To-Frame Tracking in this phantom, especially on sequence 1.

A comparison of the registration accuracy of Frame-To-Frame Tracking and Location Search in the bolus phantom is shown in Fig 11.

#### 3.2.1 Frame-To-Frame Tracking

The average MAD over all registration frames was 1.28 ± 1.04 cm, and the median MAD was 0.87 cm. Performance differed between the two video sequences, with an average MAD of 1.93 ± 1.16 cm for sequence 1 and 0.72 ± 0.43 cm for sequence 2. The individual median MADs were 1.52 cm and 0.58 cm, respectively. The reason for the difference is that the virtual endoscope became lost before reaching the bolus in sequence 1, resulting in very large MADs for the first two registration frames. However, it happened to get a view of the bolus between the second and third registration frames, resulting in smaller errors for the rest of the sequence (see Fig 11). Seven frames from video sequence 1 and one frame from video sequence 2 failed, meaning that the bolus was not visible at all in the virtual image rendered at the registered coordinates. Omitting these frames reduces the overall average MAD to 0.84 ± 0.43 cm and the median MAD to 0.67 cm.

#### 3.2.2 Location Search

The average MAD over all registration frames was 0.29 ± 0.15 cm, and the median MAD was 0.24 cm. There was no difference in performance between the two video sequences, and no frames failed.

### 3.3 Patients

The patient videos contained several unfavorable characteristics that were not present in the phantom videos. The motion of the endoscope was not as smooth and controlled, which led to erratic jumps in the camera position and more dramatic changes in lighting as the camera moved close to the walls and the image sensor adjusted its gain. The presence of saliva and other fluids creates specular reflections that were not reproduced in the virtual endoscopic images, and these fluids caused blurring when they came into contact with the camera. Finally, the anatomy of the airways of the head and neck is not rigid, which led to differences between the structures seen in the endoscopic video and in the virtual endoscopic images. Some of these differences are transient effects caused by muscle motion that were largely avoided by rejecting unsuitable registration frames, but it was evident upon visual inspection that the surfaces were not identical in the two modalities, even in the absence of apparent muscle motion. These unfavorable characteristics exacerbated the weaknesses of Frame-To-Frame Tracking, which was never able to make it through the nasal cavity before the virtual endoscope became lost. Due to these failures, only the results of the Location Search technique are presented in the following sections.

#### 3.3.1 Patient 1

Of the 38 registration frames tested for patient 1, ground truth coordinates were obtained via camera resectioning for 15 from video 1 and 12 from video 2. For the rest of the frames, camera resectioning was not possible. This occurred for a number of reasons. In the nasal cavity and superior pharynx, it was difficult to identify distinct features for corresponding points on the video frames and virtual endoscopic images. In the inferior pharynx and larynx, obscured or close-up views of the anatomy prevented the selection of an adequate number of corresponding points. Finally, there were also several frames where anatomical differences between the video and the surface mesh were sufficiently large to prevent a good resectioning.

**Fig 12:**
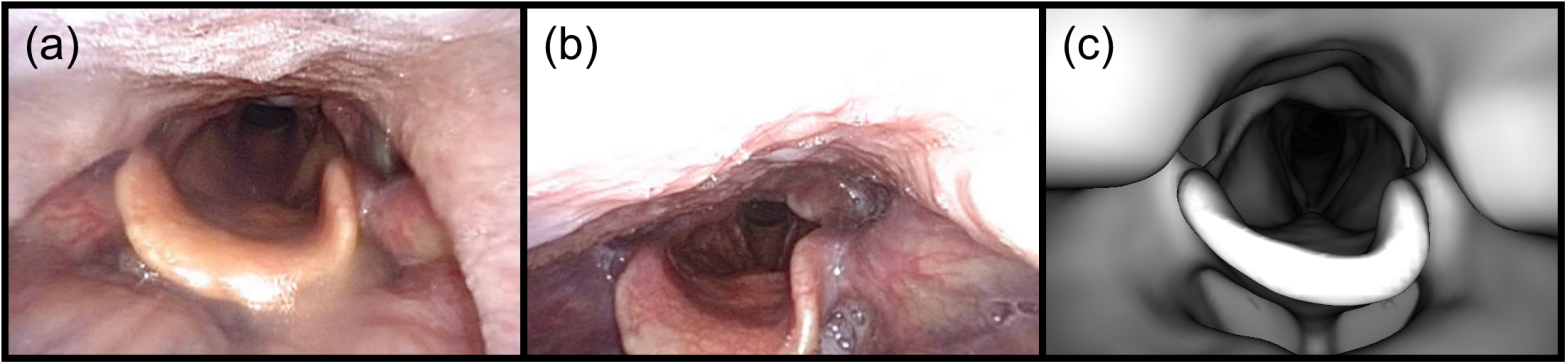
Structural differences in the pharynx between the two endoscopic examinations for patient 1. (a) A frame from video 1 showing the epiglottis and glottis. (b) A similar frame from video 2. The pharynx appears narrower, and the posterior wall of the pharynx (at the top of the image) produces a large bright area in many of the registration frames that could not be reproduced in the virtual endoscopic images. (c) A virtual endoscopic image showing the epiglottis and glottis. The surface mesh appears wider than the pharynx in both videos, but the differences are particularly apparent when compared to video 2.

**Fig 13:**
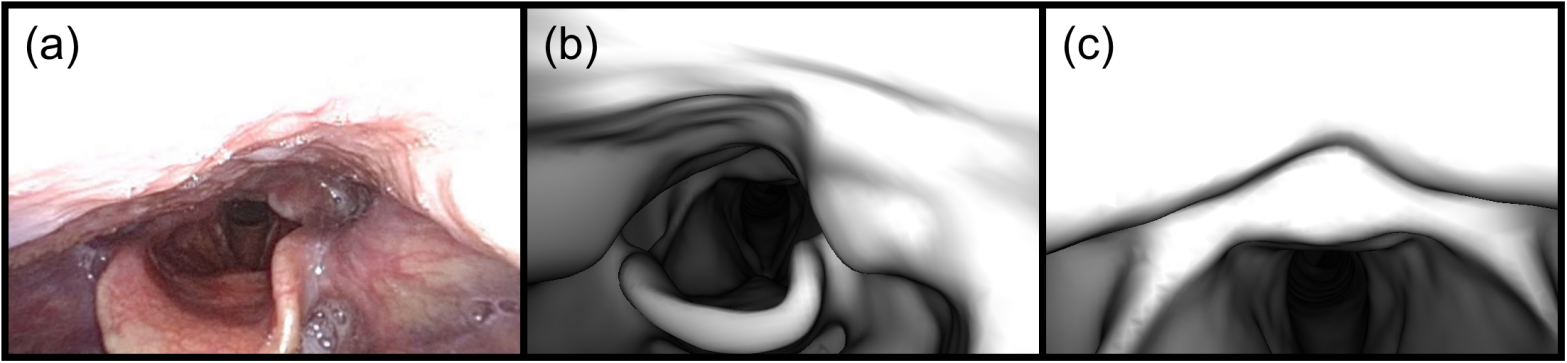
Registration failure caused by structural differences between the endoscopic video and the virtual endoscopic images. (a) A registration frame from video 2 showing the epiglottis and glottis. (b) The virtual endoscopic image rendered at the ground truth coordinates obtained via camera resectioning. (c) The virtual endoscopic image rendered at the registered coordinates obtained via Location Search. The similarity measure matched the large bright area in the registration frame to the bright region seen in this virtual image inferior to the epiglottis. The distance between the ground truth and registered coordinates was 3.66 cm.

For these 27 frames, the average distance between the measured and ground truth endoscope positions was 1.88 ± 1.41 cm, and the median distance was 1.51 cm. Performance differed between the two videos, with an average distance of 1.22 ± 0.79 cm for video 1 and 2.69 ± 1.57 cm for video 2. The individual median distances were 0.91 cm and 2.92 cm, respectively. The main reason for the difference in performance appears to be a difference in the posterior wall of the pharynx. The pharynx was narrower in video 2, with the posterior wall closer to the epiglottis (see Fig 12). This produced a large bright area at the top of the registration frames that could not be reproduced in the virtual images (see Fig 13).

Of the 27 registration frames with ground truth coordinates, three from video 1 and seven from video 2 were subjectively classified as failures, meaning that the virtual image rendered at the registered coordinates did not match the anatomy in the registration frame at all. If these frames are omitted, the average distance between the measured and ground truth endoscope positions is reduced to 0.98 ± 0.53 cm, and the median is reduced to 0.82 cm.

#### 3.3.2 Patient 3

**Fig 14:**
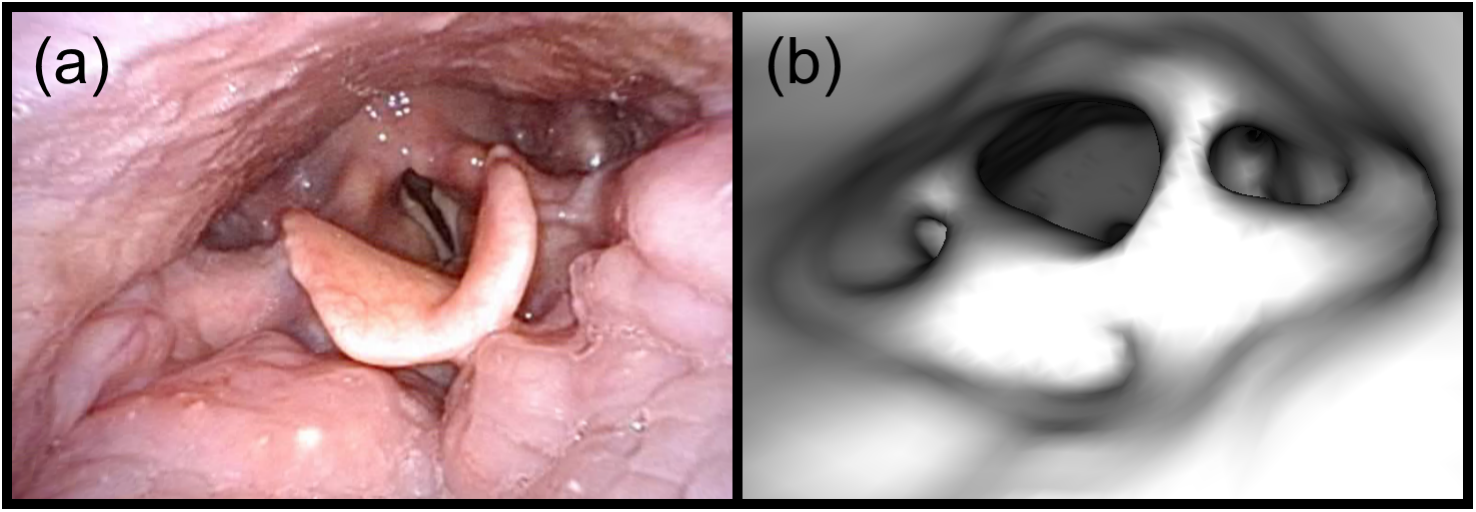
Virtual endoscopic images lacked anatomical detail in patient 3. (a) An endoscopic video frame showing the epiglottis and glottis. (b) A virtual endoscopic image in the same region. The epiglottis is poorly defined and attaches to the posterior wall at the top of the image. The region in front of the epiglottis has very little structure in the virtual image as well. The glottis is also poorly defined, and, unlike in the video frame, it is offset anteriorly and can barely be seen behind the epiglottis.

**Fig 15:**
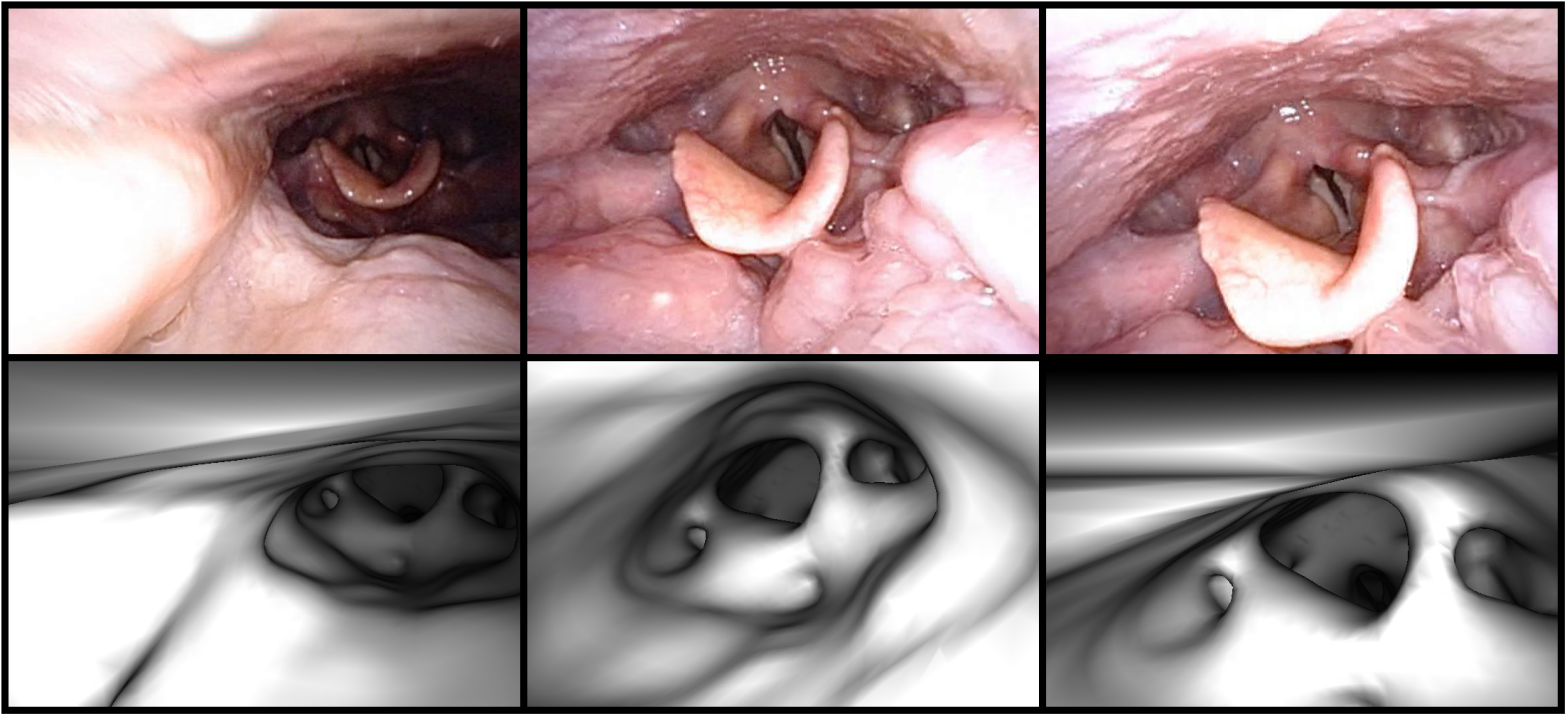
Three examples of successful registrations in patient 3. The top row shows three registration frames, and the bottom row shows the corresponding virtual endoscopic images rendered at the registered coordinates obtained via Location Search. None of these is a perfect match, and the virtual endoscope appears to be farther from the epiglottis in the virtual images than the camera was in the video frames. However, even with the lack of detail in the surface mesh, Location Search was able to find reasonable matches for these video frames.

Camera resectioning was not possible for patient 3’s registration frames, so ground truth endoscope coordinates could not be obtained. There were two reasons for this failure, which are illustrated in Fig 14. The first is that the surface mesh, and hence the virtual endoscopic images, contained very little anatomical detail. Both scans were acquired with the same parameters on the same scanner, so it is unclear why the mesh had this appearance. One possibility is that muscle motion during CT acquisition caused a blurring effect which carried over into the mesh. The second reason ground truth coordinates could not be obtained is that there was an anterior offset in the position of the glottis between the video and the virtual endoscopic images. These characteristics caused the resectioning process to be poorly conditioned, such that the coordinates obtained were outside of the surface mesh or simply did not match the video frame. Despite these weaknesses, Location Search was able to find reasonable endoscope coordinates for several of the registration frames. Three of these are illustrated in Fig 15.

## 4. Discussion

### 4.1 Summary

This study developed and investigated Location Search, a new technique to register endoscopic video to CT, which does not require prospective tracking of the endoscope with physical sensors during the examination. This technique uses physical constraints on endoscope position and orientation to search the airways of the head and neck efficiently in order to find the endoscope coordinates for a given video frame by maximizing the similarity between the video frame and virtual endoscopic images. The performance of Location Search was assessed in both rigid phantoms and patients, and compared to that of a previously-established technique, Frame-To-Frame Tracking.

In the phantoms, Location Search had better registration accuracy overall, especially for mapping the bolus material from video frames to CT space. Frame-To-Frame Tracking suffered from large errors in both phantoms when the virtual endoscope became lost and could not recover. Location Search failed on some frames as well, but unlike Frame-To-Frame Tracking, a failure in one frame does not affect any subsequent frames. In patients this difference between the techniques was especially apparent. Frame-To-Frame Tracking was unable to make it through the nasal cavity, and no frames were successfully registered. Location Search was able to successfully register many frames in the patient videos, but its accuracy suffered in the presence of unfavorable conditions including muscle motion and structural differences between the anatomy seen in the endoscopic video and that seen in the virtual endoscopic images.

### 4.2 The impact of manual inputs

The Location Search technique requires manual input to create the possible endoscope path through the volume. This was done by selecting a small set of points from the nasal cavity past the glottis on a sagittal CT slice. This will introduce some variability between users, but it is important to note that Location Search does not depend on the similarity between the possible path and the actual path taken in the recorded video. No effort was made to replicate the actual path when creating the paths used in this study.

The path is used to assign virtual endoscope view directions when slicing the surface and when initializing the simplex optimizations. It is possible that a highly-convoluted path drawn by a user could cause inadequate sampling of the volume if the slices are at odd angles, or could initialize the virtual endoscope with a view direction that causes the simplex to find a false optimum. However, the possible endoscope path is meant to be a simplified approximation like the one illustrated in Fig 8. If the path is drawn in this manner, it is unlikely that inter-user variability will impact the registration.

Manual input was also used to identify point correspondences to obtain ground-truth virtual endoscope coordinates via camera resectioning for patient images. Effort was made to select these points covering a range of distances from the camera, which improves the conditioning of the resectioning and should reduce the impact of inter-user variability. However, the impact of inter-user variability was not quantified for this or the path creation. The purpose of this study was to explore the feasibility of endoscopy-CT image registration, but if the Location Search technique is to be used clinically, these manual inputs must be standardized, or automated if possible.

### 4.3 Computation times

One drawback of the Location Search technique is its computational complexity. The creation of the virtual endoscope path and the creation of the set of seed points by slicing the surface and k-means clustering (steps 2 and 3 in section 2.3.2) need to be done only once for a given phantom or patient, but the coarse search over all of the seed points and the subsequent fine search (steps 4–6 in section 2.3.2) must be done for each frame. This process required about 15 minutes per frame in the current implementation, which is written in the Python programming language. This is certainly a practical limitation, but there are many aspects of this algorithm that can be optimized to reduce the computation time. The most salient target is parallel computing for the coarse search, as the optimization at each seed point is independent of that at all other seed points.

### 4.4 Failure modes

Only three patients were analyzed in this study, but the diversity of the cases in this small set offers insight into the feasibility of endoscopy-CT image registration. The registration techniques could not be tested on patient 2, whose large tumor made virtual endoscopy very difficult. This suggests that there is a subset of head and neck cancer patients for whom endoscopy-CT registration is simply not possible. Both registration techniques were tested on patients 1 and 3, but there was a considerable difference in the appearance of their virtual endoscopic images, with very little anatomical detail visible for patient 3. The CT scans were acquired with the same parameters on the same scanner, so the source of this discrepancy is unknown. There may have been muscle motion during CT acquisition that caused a blurring effect on the surface mesh for patient 3. These endoscopy-CT registration techniques rely on virtual endoscopy, and if sufficient anatomical detail cannot be retained in the surface mesh, registration based on image similarity is not possible.

When using Location Search, the virtual endoscope cannot become lost as it can with Frame-To-Frame Tracking. There is typically a local optimum in the similarity measure when the virtual endoscope is near the correct location for a given registration frame, but Location Search can fail when that optimum is not the global optimum. This occurred for two frames in the marker phantom (see Fig 10 and section 3.1.2) and for numerous frames in the patient videos (see Fig 13). In the patient images, it was often the case that the second or third best result from the coarse search over all seed points was a good match for the registration frame. These good and bad matches are not challenging to identify, but any additional requirement of manual input will reduce the utility of endoscopy-CT registration in routine clinical practice.

### 4.5 Local optima in the Nelder-Mead simplex optimization algorithm

Both Frame-To-Frame Tracking and Location Search use the Nelder-Mead simplex to optimize virtual endoscope coordinates to find the best match for the registration frame. This method was chosen because it does not require any gradient computation, which is not possible in this scenario. However, it is a local optimization technique that can only find the nearest optimum. This is not a problem with Frame-To-Frame Tracking, because in a 30 frames-per-second video, the next frame is always going to be close enough to the previous solution that local optimization is sufficient. But when trying to register a frame with no nearby initial guess for the virtual endoscope coordinates, a global search is required. Location Search accomplishes this by treating the position and orientation components of virtual endoscope coordinate space separately. The slicing and clustering of seed points samples the position space of the entire volume. The manually-drawn possible endoscope path eliminates the need for a global search of orientation space, because it is used to initialize the virtual endoscope’s view direction sufficiently close to the optimum. So even though a local optimization algorithm is used in both techniques, Location Search effectively performs a global optimization by using many start points that sample the entire volume. Its performance depends on the seed point sampling being dense enough that the optimum is not missed.

As mentioned in section 4.3, there were several patient video frames for which Location Search failed, but the second or third best result from the seed optimization was a good match for the registration frame. These represent a failure of the similarity measure to correctly distinguish the anatomy in the registration frame, rather than the simplex getting stuck in a local optimum. However, it may be advantageous to modify Location Search to perform the initial coarse search using a global optimization technique such as simulated annealing.

### 4.6 Anatomical changes caused by patient positioning

Virtual endoscopy surface meshes were created from planning CTs, which are acquired in the supine position with the patient’s head, neck, and shoulders positioned with a thermoplastic mask. The patient’s head is typically tilted back to keep it out of the treatment beam. Routine endoscopic examinations are performed with the patient seated in a chair, and the patient is not positioned in any particular way. One weakness of the methods presented in this study is that it is likely that the different positions introduce anatomical differences between the endoscopic video and the virtual images. These differences could prevent successful registration when the virtual image cannot match the appearance of the video frame at the correct coordinates, and could introduce errors when mapping video frames to CT even when registration is successful. It is possible that patient positioning is the source of the anatomical differences discussed in sections 3.3.1 and 3.3.2 and in Figs 12 and 13. Without a patient set including supine and upright CTs, it will be difficult to fully understand the impact of patient positioning. A possible solution to this problem is to allow the surface mesh to deform to match the appearance of the anatomy in the endoscopic video, but validation will remain a challenge. Another option that would reduce positioning differences is to perform the endoscopic examination with the patient in the supine position using the thermoplastic mask. This would not cause undue burden on the patient, but it is a change to standard clinical practice. The thermoplastic masks are affixed to mounts on the couch of a CT scanner or linear accelerator, and scheduling time to use this equipment could pose a logistical challenge in busy clinics. Furthermore, development of image registration for seated-position endoscopy is appealing because it could be used in scenarios where supine endoscopy is not available, such as retrospective analysis of archived video or endoscopic examinations performed at outside institutions.

### 4.7 Future directions to improve Location Search

The accuracy of Location Search on patient images in this study is not sufficient for clinical use. However, there are many aspects of this technique that remain to be optimized. One of these aspects is the virtual endoscopy lighting model, which is very important for image similarity calculations. The model used in this study is very simple. It does not include reflection of light from the walls of the airway, and it uses a static intensity, while the endoscope’s image sensor constantly adjusts its gain as the endoscope moves closer and farther from the walls. Incorporating these effects into the lighting model may improve registration. Another target for future work is calculating image similarity using certain regions of the video frames and virtual images, rather than the entire images. This may be able to prevent the false matches described in section 4.3, and it would also improve computation times by reducing the number of pixels considered when calculating image similarity.

## 5. Conclusions

Both Frame-To-Frame Tracking and Location Search can accurately register endoscopic video to CT. Location Search is more robust against unfavorable video characteristics that are inevitably present in endoscopic examinations, but that robustness comes at the cost of increased computational complexity. Both techniques were tested independently in this study, and future efforts will draw on the strengths of both to create a more complete endoscopy-CT registration framework. Many aspects of these techniques remain to be optimized, including image processing details, automation to reduce the need for user input, and parallelization to minimize computation time. The results of this study show that endoscopy-CT image registration without prospective physical tracking of the endoscope is accurate on rigid phantoms and feasible on patient images, but more development is required and more data must be collected before this new technique can be translated into clinical practice.

## Supporting information

S1 Dataset The raw data used in this study. This spreadsheet contains three sheets. “Patient frame coordinates” contains the measured and ground-truth virtual endoscope coordinates for the patient videos. “Marker position errors” contains the distances between measured and ground-truth marker positions in the marker phantom. “Bolus contour errors” contains the mean absolute distances between the measured and ground-truth bolus contours in the bolus phantom.
(XLSX)
